# Real-time label-free three-dimensional invasion assay for anti-metastatic drug screening using impedance sensing

**DOI:** 10.3389/fphar.2024.1387949

**Published:** 2024-06-11

**Authors:** Kai Ding, Hailong Li, Qian Xu, Yongmei Zhao, Kaikai Wang, Tianqing Liu

**Affiliations:** ^1^ School of Pharmacy, Nantong University, Nantong, China; ^2^ NICM Health Research Institute, Western Sydney University, Westmead, NSW, Australia

**Keywords:** tumor metastasis, tumor spheroid, biosensing, drug screening, drug development

## Abstract

Tumor metastasis presents a formidable challenge in cancer treatment, necessitating effective tools for anti-cancer drug development. Conventional 2D cell culture methods, while considered the “gold standard” for invasive studies, exhibit limitations in representing cancer hallmarks and phenotypes. This study proposes an innovative approach that combines the advantages of 3D tumor spheroid culture with impedance-based biosensing technologies to establish a high-throughput 3D cell invasion assay for anti-metastasis drug screening through multicellular tumor spheroids. In addition, the xCELLigence device is employed to monitor the time-dependent kinetics of cell behavior, including attachment and invasion out of the 3D matrix. Moreover, an iron chelator (deferoxamine) is employed to monitor the inhibition of epithelial–mesenchymal transition in 3D spheroids across different tumor cell types. The above results indicate that our integrated 3D cell invasion assay with impedance-based sensing could be a promising tool for enhancing the quality of the drug development pipeline by providing a robust platform for predicting the efficacy and safety of anti-metastatic drugs before advancing into preclinical or clinical trials.

## Introduction

Tumor metastasis poses a significant challenge in cancer treatment, with the complex biological processes involved making it difficult to study (Lyden et al., 2022). However, it is very challenging to screen anti-cancer drugs against tumor metastasis *in vitro*, mostly because there is a lack of effective tools for drug development that can be a good mimic for tumor metastasis (Wan et al., 2020; Collins et al., 2021). As a result, it is difficult to identify the potential compounds to treat tumor metastasis, leading to poor prediction of the drug’s therapeutic efficacy and safety before preclinical or clinical trials. This increased the cost of drug development due to the failure of the anti-cancer drug candidates to go to market. Therefore, establishing an advanced, high-throughput platform for anti-metastatic drug screening is in high demand to increase the pipeline quality.

Conventional 2D cell invasion assays, such as the well-established scratch assay, are widely acknowledged as the gold standard methods for studying invasive processes (Bouchalova and Bouchal, 2022). However, it has been reported that 2D cancer cell culture could not represent some hallmarks of cancer in the expression of some phenotypes, while cancer cells cultured in a 3D environment undergo significant phenotypical changes, such as epithelial–mesenchymal transition (EMT), due to 3D cell–cell interactions, the presence of complex extracellular matrix, and the physiological spatial heterogeneity (Liu et al., 2014; Liu et al., 2014; Golinelli et al., 2021; Leineweber, 2023; Zhu et al., 2023). An improved assay to mimic tumor invasion using *in vitro* models combines 3D with 2D cell culture by placing multicellular tumor cell spheroids either on a conventional cell culture dish or on collagen-coated surfaces (Manini et al., 2018; Weydert et al., 2020; Rodrigues et al., 2021). After the spheroids are attached to the surface, the cancer cells start to migrate on the dish surface opposite to the center of the spheroids in a scattering manner or invade toward the collagen as the extracellular matrix. Cell invasion behavior can be monitored using microscopes over a certain period of time or recorded at endpoints. The invasion of cancer cells toward the surfaces from the spheroids can closely mimic tumor aggressiveness and response to biological or drug conditions (Zuela-Sopilniak and Lammerding, 2019). Therefore, this invasion assay has been used for anti-migratory effects of drugs or biomolecules on 3D tumor tissues.

**FIGURE 1:**
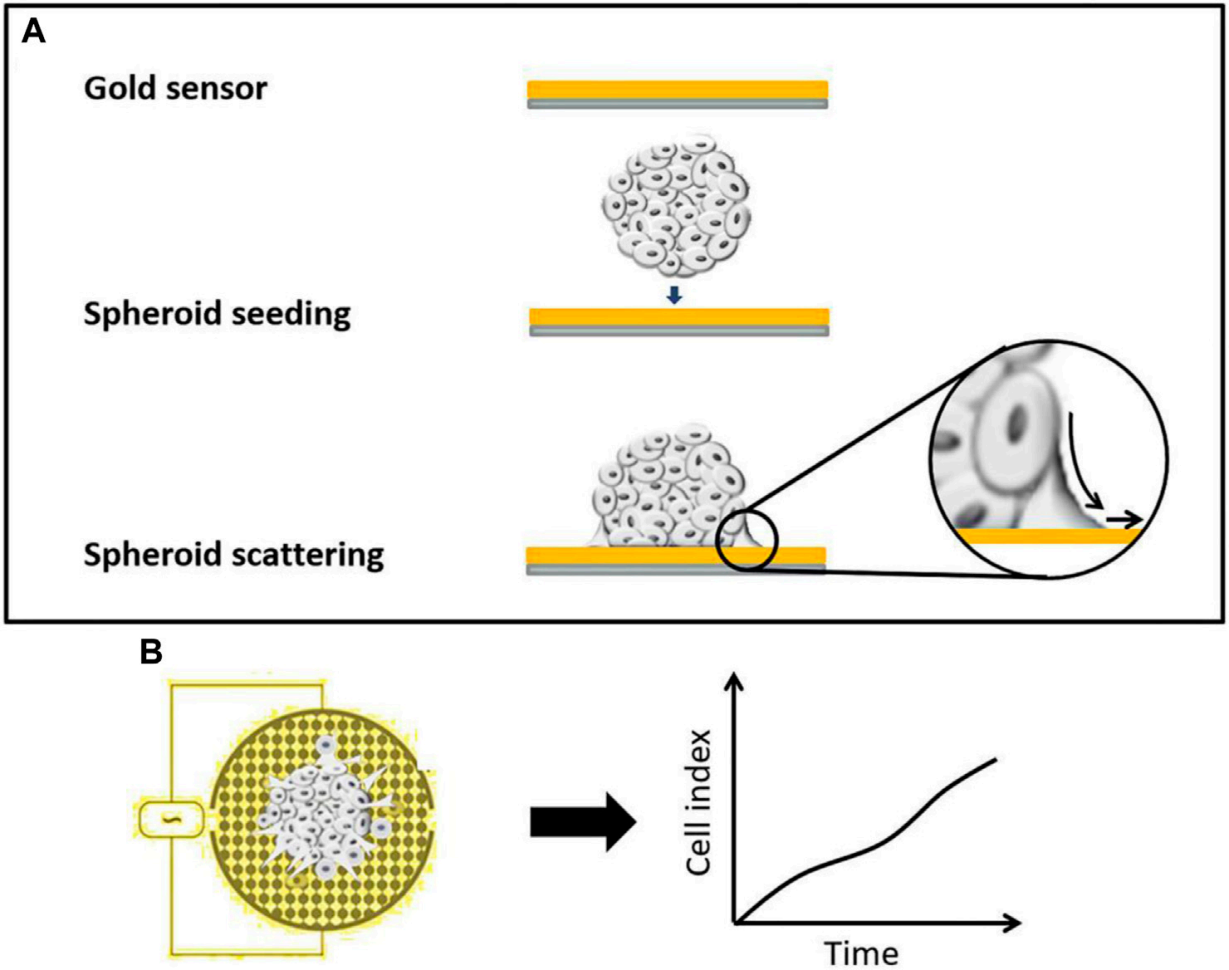
Schematic of the impedance-based spheroid spreading assay.

Impedance-based cell substrate sensing has emerged as a powerful, real-time, non-invasive, and label-free approach to assess various cell events, such as attachment, adhesion, proliferation, and invasion (Ramasamy et al., 2014; Zhang et al., 2021; Chen et al., 2023). Traditionally, the standard method for impedance-based cell sensing is to culture cells as a monolayer on the gold electrode, which can detect the changes in resistance caused by the cell behavior responding to the treatment (Mojena-Medina et al., 2020; Sagir et al., 2022). However, there is a lack of impedance-based cell assays developed to study cell invasion using 3D tissue models. Here, we present an integrated strategy for combining 3D tumor spheroid culture with commercially available impedance-based biosensing technologies in order to develop a 3D cell invasion assay for high-throughput anti-metastasis drug screening (Figures 1A,B). Multicellular tumor spheroids are cultured in a concave microwell device and then transferred to the impedance sensor (Tevlek et al., 2023). Cell attachment to the substrate and cell invasion out of the 3D matrix can be monitored over time by recording time-dependent kinetics of cell behavior using the xCELLigence device (Yan et al., 2018). The system is established to evaluate the anti-migratory effects of drugs on 3D tumor spheroids using a commercially available technology. The data are compared with parallel data obtained from conventional 2D and 3D observation-based invasion assays using cell lines, which are known to have different invasion capabilities, to confirm the reliability of this method. Furthermore, the application of the iron chelator (deferoxamine) adds a specific dimension to the study. The treatment-induced inhibition of EMT can be monitored using this 3D spheroid assay across different types of tumor cells. This comprehensive approach not only advances our understanding of cell invasion in 3D environments but also provides a valuable tool for high-throughput screening of anti-metastasis drugs in a physiologically relevant context.

## Materials and methods

### Cell culture

Two cell lines with different tumor metastasis status were used in this study. Human adenocarcinomas MCF-7 and MDA-MB-231 were purchased from the American Type Culture Collection (ATCC, United States). MCF-7 and MDA-MB-231 cells were grown in a complete medium consisting of DMEM supplemented with 10% (v/v) FBS in a humidified incubator at 37°C in 5% CO_2_ and at 100% humidity. Cells were maintained by a once-weekly passage using trypsin/EDTA.

### Cell seeding and tumor spheroid formation

Tumor spheroids were prepared using our previously developed method (Liu et al., 2014). The microwell devices were equilibrated with cell culture medium for 30 min before cell seeding. Cancer cell suspensions of MCF-7 or MDA-MB-231 cells (400 μL, 1 × 10^5^ cells/mL) were seeded into the devices. Cell sedimentation in the microwells was aided by gentle vibration. The culture medium was removed every 2 days and refilled with fresh medium. The microwell devices were typically kept in an incubator for several days to enable the formation of dense multicellular tumor spheroids.

### 2D cell scratch assay monitored using time-lapse microscopy

Cancer cells were seeded on the 96-well plates with a cell seeding density of 200 μL, 1 × 10^6^ cells/mL, and attached to the surface overnight. After the cells reached confluence, a line was drawn on the cell monolayer using a cell scraper, and a scratched region was created. Cell invasion over the scratched region was monitored over 24 h (Huang et al., 2020; Pijuan et al., 2019). The distance of the recovery over time was monitored using a time-lapse microscope with a stage-top incubator.

### 3D spheroid invasion assay

Cells were seeded into agarose microwells to form 3D tissue. The formed spheroids were harvested from the microwell devices and manually transferred to the collagen-coated E-plate. The cell motion and the invasion of 3D spheroids were tracked using a time-lapse microscope equipped with impedance-sensing technology.

### Antiproliferative and metastasis- predictive drug treatment

An iron chelator, deferoxamine (DFO), was dissolved and diluted using a culture medium as the treatment. The anti-proliferative effects of the drug on different cancer cells were measured after 72 h of incubation at 37°C (Sandoval-Acuña et al., 2021; Wijesinghe et al., 2021). The proliferation of the MCF-7 and MDA-MB-231 cells was first monitored using an xCELLigence System (Roche, Penzberg, Germany). The cell index, defined based on the cell adhesion rate, was recorded throughout the incubation and treatment periods. IC_50_ values were obtained directly from the measurement. The drug response was evaluated using both 2D cell scratch assays monitored with a time-lapse microscope and 3D spheroid invasion assays were monitored using a time-lapse microscope and impedance-sensing equipment.

### Western blot of EMT biomarkers

The proteins were extracted from the 2D cells and 3D tumor spheroids using RIPA buffer containing a protease inhibitor, a phosphatase inhibitor, and phenylmethanesulfonyl fluoride (PMSF). They were separated with SDS-PAGE gel and transferred onto a polyvinylidene fluoride (PVDF) membrane. After being blocked in 5% BSA for 2 h at room temperature, the membranes were incubated with primary antibodies for EMT markers (vimentin, E-cadherin, and N-cadherin) and β-actin at a 1:1000 dilution in 5% BSA at 4°C overnight. After being washed three times with TBST every 15 min, the membranes were incubated with the secondary antibody at room temperature for 2 h. The blots were visualized using an ECL chemiluminescence kit. β-Actin was used as a loading control, and Image-Pro software (National Institutes of Health, Bethesda, MD, United States) was used for the densitometric analysis of the bands.

## Results and discussion

### 2D cell scratch assay using cancer cells with different metastatic statuses

**FIGURE 2:**
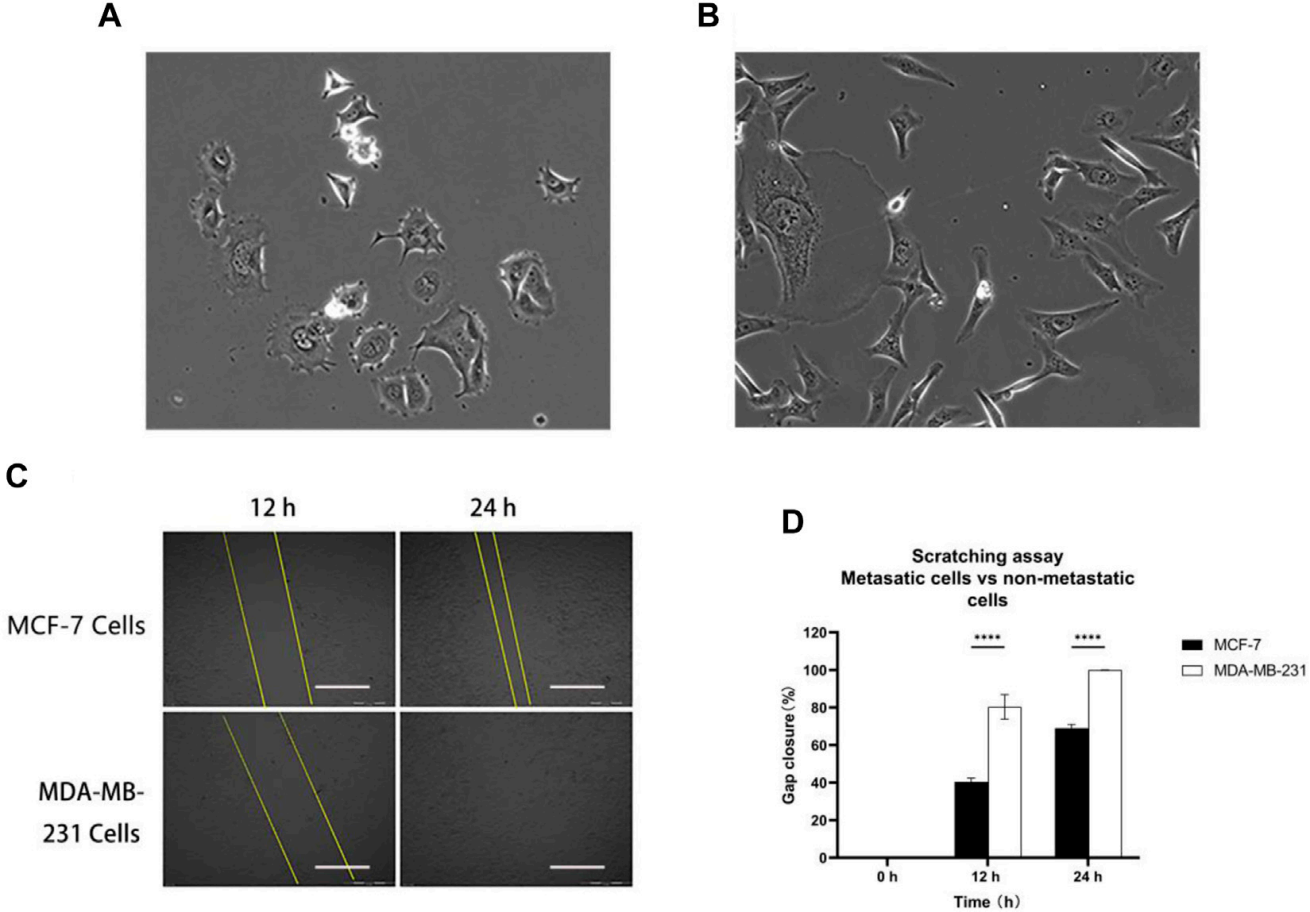
Metastatic cells vs. non-metastatic cells: **(A)** MCF-7 and **(B)** MDA-MB-231 cell morphology; **(C)** and **(D)** MCF-7 and MDA-MB-231 cells were scratched and monitored over 24 h. Scale bar, 0.5 mm. Then, wound healing was analyzed using ImageJ software. Data are expressed as mean ± SD of three independent replicates. ****p < 0.0001 *versus* the 2D group.

Two distinct metastatic statuses of human breast cancer cells, namely MCF-7 cells (Figure 2A) and MDA-MB-231 cells (Figure 2B), were chosen for the 2D cell scratch assay. MDA-MB-231 cells, which are characterized by their spindle shape, estrogen receptor (ER) negativity, adherent growth, and robust invasion and metastatic capabilities, were selected as representatives of highly invasive human breast cancer cells. On the other hand, MCF-7 cells, which are estrogen receptor-positive breast cancer cell lines, exhibit lower invasiveness compared to MDA-MB-231. The healing process in the MCF-7 cell line showed only 40% closure at the 12th hour and approximately 70% at the 24th hour, indicating incomplete healing. In contrast, the MDA-MB-231 cell line exhibited 80% wound closure at the 12th hour and complete healing at the 24th hour (Figures 2C,D). These findings suggest that MDA-MB-231 cells are highly invasive, while MCF-7 cells exhibit lower invasiveness. Importantly, the observed experimental outcomes align with the existing literature data, supporting the reliability and consistency of our results (Izdebska et al., 2021).

### Verification of the epithelial–mesenchymal transition (EMT) induced by 3D cell culture

**FIGURE 3:**
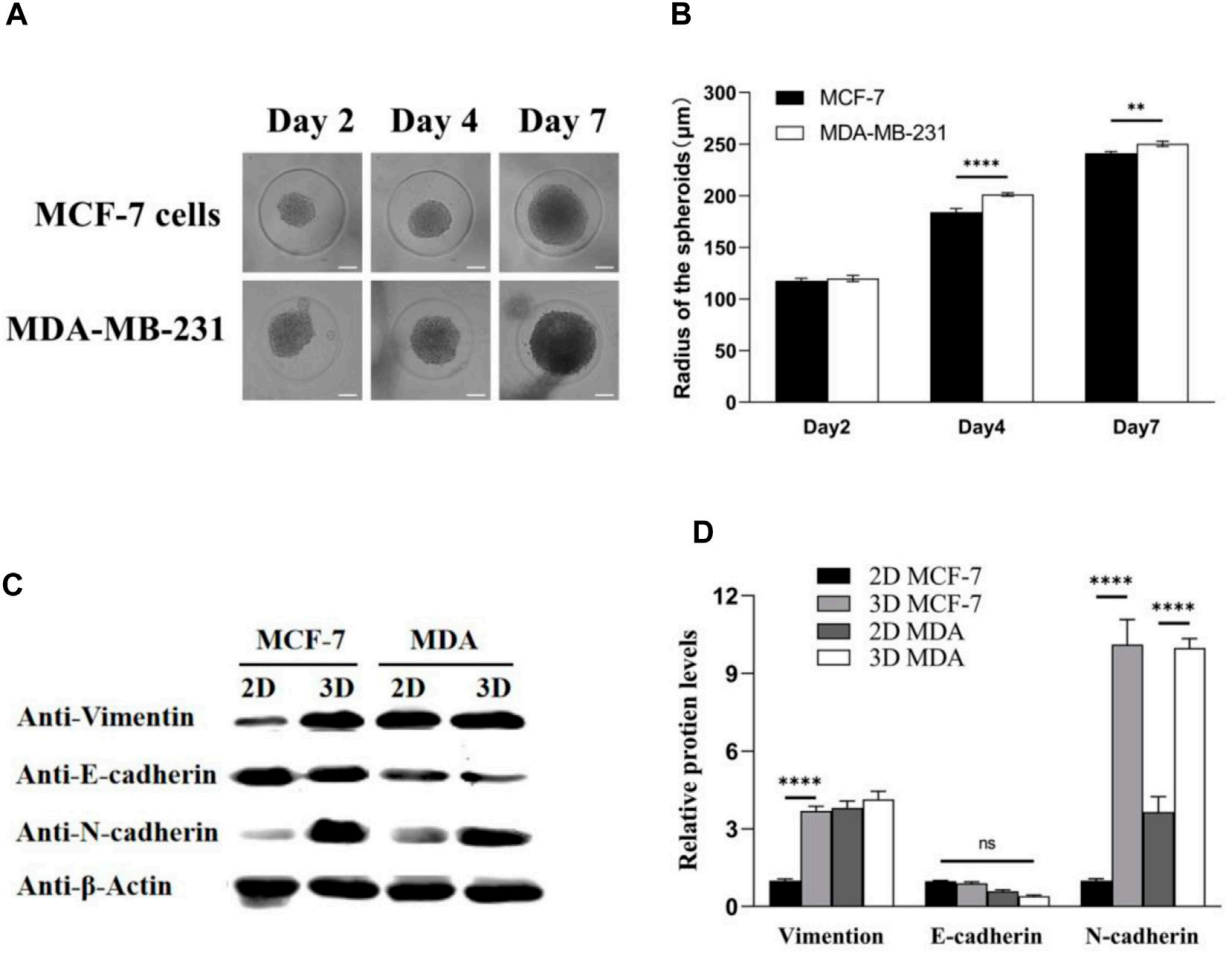
MDA-MB-231 spheroids significantly contribute to the expression levels of EMT biomarkers compared with 2D culture. **(A)** MCF-7 and MDA-MB-231 spheroids in microwells; **(B)** spheroid radius was analyzed by Image-Pro Analyzer software; and **(C,D)** Western blotting analysis on the expression of EMT biomarkers: vimentin, E-cadherin, and N-cadherin. β-Actin was used as the internal reference. Scale bar, 100 μm. (**p < 0.01, ****p < 0.0001, N = 3, six spheroids per group).

Furthermore, tumor spheroids were prepared by culturing MDA-MB-231 and MCF-7 cells in microwells at a concentration of 1 × 10^5^ cells/device. The growth of multicellular tumor spheroids was monitored using a time-lapse microscope, and changes in spheroid size were quantified using Image-Pro Analyzer software. Experimental findings revealed that, by day 7, the radius of MDA-MB-231 tumor spheroids was approximately 250 μm, surpassing that of MCF-7 tumor spheroids (Figure 3A). Real-time tumor images further illustrated the larger size of MDA-MB-231 cell-derived spheroids compared to those derived from MCF-7 cells (Figure 3B). Concurrently, protein extraction from both 2D and 3D cultured MDA-MB-231 and MCF-7 cells enabled a comparison of the expression of epithelial–mesenchymal transition (EMT)-related proteins using Western blot analysis (Figures 3C, D). In 2D culture, vimentin protein expression in MDA-MB-231 cells exceeded that in MCF-7 cells, suggesting a higher invasiveness of MDA-MB-231 cells. Interestingly, 3D culture led to a significant increase in vimentin expression in MCF-7 cells, indicating an elevation in the invasiveness of the initially less-invasive tumor. Notably, the absence of E-cadherin expression is indicative of EMT, and MDA-MB-231 cells exhibited lower E-cadherin expression compared to MCF-7 cells, reinforcing the higher invasiveness of MDA-MB-231 cells. Moreover, the expression of E-cadherin in 3D tumor spheroids for both cell types was lower than that in 2D culture, suggesting that 3D culture increased the invasiveness of tumor cells. Furthermore, N-cadherin, which is associated with increased motility and invasiveness, exhibited elevated expression in 3D-cultured tumor spheroids compared to 2D culture. In summary, 3D culture promoted the enhanced epithelial–mesenchymal transition in tumors. Tumor spheroids cultured in 3D more closely resembled real tumors compared to their 2D counterparts, making them more conducive to subsequent experimental analyses.

### Comparison of the 3D spheroid scattering assay

There are limitations associated with 2D cell culture. Studies have indicated that 2D cancer cell culture fails to fully capture certain characteristics of cancer phenotypes. In contrast, cancer cells cultured in a 3D environment undergo substantial phenotypic changes, such as epithelial–mesenchymal transition (EMT), owing to 3D cell–cell interactions, the presence of a complex extracellular matrix, and physiological spatial heterogeneity (Leggett et al., 2021). To address these limitations, we employ a method that combines *in vitro* 3D-cultured tumor spheroids with an impedance sensor capable of monitoring real-time changes in cell morphology, proliferation, and differentiation quantitatively.

**FIGURE 4:**
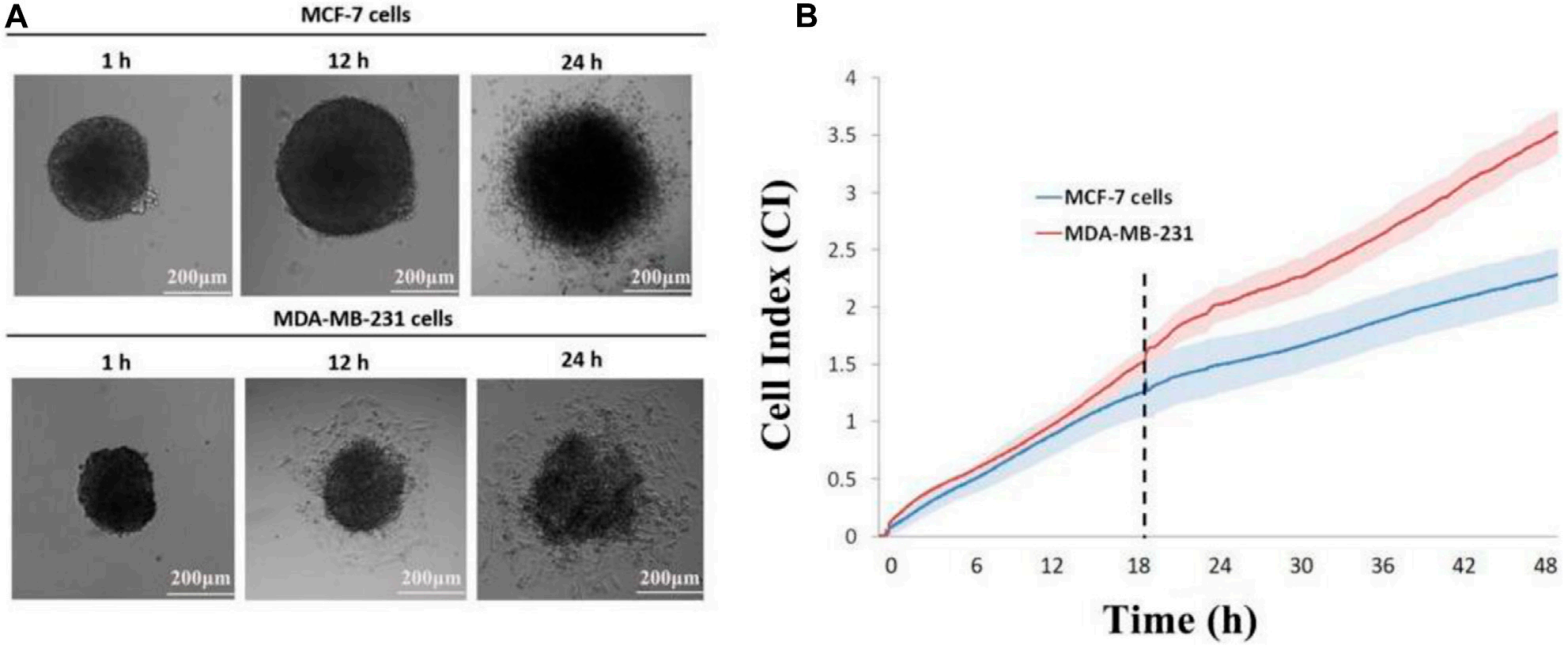
Impedance sensing of both cell lines. **(A)** Morphological changes in MCF-7 and MDA-MB-231 spheroids on E-plate; **(B)** real-time monitoring of cell index changes in tumor spheroids using impedance sensors. Scale bar, 200 μm. (N = 3, six spheroids per group).

In this approach, multicellular tumor spheroids are cultured in a concave microwell device and then transferred onto the impedance sensor (Lee and Kim, 2021). Over the course of several hours, tumor cells spread from the spheroid onto the coated surface, and their invasion is recorded at intervals of up to 72 h. Using an inverted microscope, images are captured and subsequently analyzed using Image-Pro Analyzer software. Manual recording of the leading edge of migrating cells allows the software application to calculate the covered area, offering valuable qualitative insights into different cell invasion patterns (Figure 4B). The recorded cell index (CI) demonstrates a positive correlation with the invasive capacity of the tumor cells. Notably, MDA-MB-231 achieved the highest CI value in the invasion (without Matrigel) setting, indicating that the 3D tumor spheroid from the MDA-MB-231 cell line exhibited greater mobility than that from the MCF-7 cell line (Figure 4B). The experimental monitoring results align with the observations made through real-time imaging using a time-lapse microscope (Figure 4A).

### Inhibition of EMT using iron chelators monitored by the impedance-sensing-based 3D spheroid scattering assay

Following the experimental validation of the real-time 3D spheroid invasion monitoring capability of the impedance sensor, our objective was to utilize this sensor for tracking the inhibition of epithelial–mesenchymal transition (EMT) in 3D spheroids of distinct tumor cells through iron chelator (DFO) treatment. Initially, we assessed the impact of different concentrations of iron chelators on the dispersion of 3D spheroids by monitoring changes in cell index (CI) values. Subsequently, the selected concentration of iron chelators was applied to 3D tumor spheroids on E-plates, and impedance curves were recorded from the initiation. The addition of the iron chelator (DFO) occurred during the plateau period of the impedance curves, concurrent with the real-time documentation of the 3D spheroid states.

**FIGURE 5:**
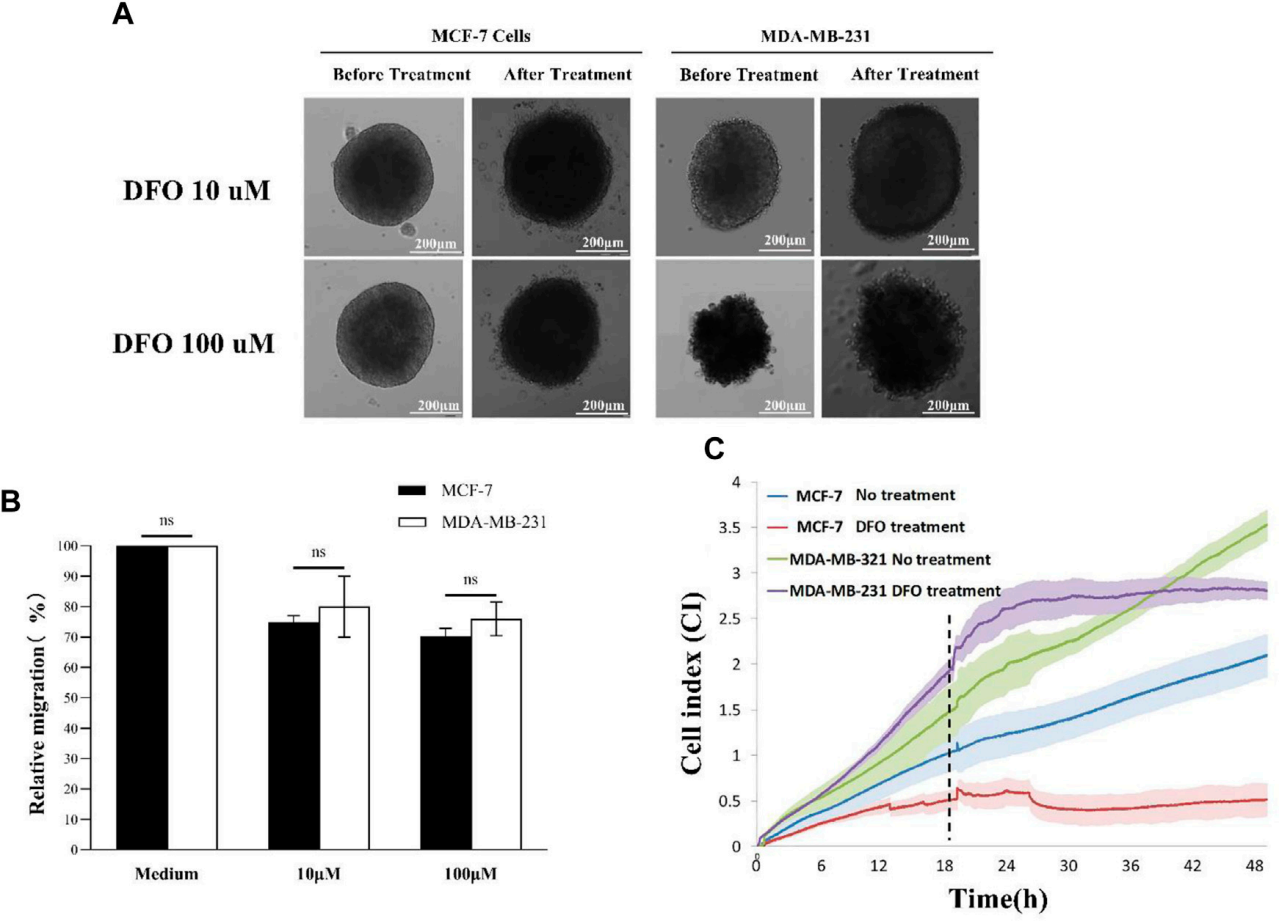
DFO treatment: **(A)** 3D scattering assay recorded using time-lapse microscopy; **(B)** relative invasion of 3D tumor spheroids; and **(C)** impedance sensing. Scale bar, 200 μm. (N = 3, six spheroids per group).

Time-lapse microscopy data revealed a diminished invasion of both MCF-7 and MDA-MB-231 spheroids, following DFO treatment, with invasion capacity decreasing in correlation with the increasing DFO concentration (Figure 5A). Consistent findings were obtained through Image-Pro Analyzer software analysis, further indicating that MCF-7 spheroids exhibited lower relative invasion compared to MDA-MB-231 spheroids, aligning with previous experimental outcomes (Figure 5B). Real-time impedance values demonstrated that the MCF-7 DFO treatment group exhibited the lowest cell index, while the MDA-MB-231-no treatment group reached the highest cell index at the 48th hour post-chelating agent administration. In the MDA-MB-231 DFO treatment group, the cell index briefly increased to around 2.5 following chelating agent administration before plateauing for a period, whereas in the no-treatment groups, the cell index continued to rise, surpassing that of the treated group after 18 h. These observations indicated that the EMT of MDA-MB-231 tumor spheroids was suppressed by iron chelator treatment. Similar trends were observed in the less-invasive MCF-7 cells (Figure 5C).

## Conclusion

In conclusion, our investigation not only confirmed the augmented expression of epithelial–mesenchymal transition (EMT)-related biomarkers within 3D tumor spheroids when compared to their 2D cultured counterparts, as validated by Western blot analysis but also extended its scope to encompass the real-time monitoring of cell invasion using the impedance sensor system. This multifaceted approach provided a better understanding of the dynamic changes in cellular behavior under three-dimensional conditions. Furthermore, we successfully demonstrated the efficacy of the impedance sensor system in verifying the inhibitory effects of the DFO drug on the invasion of 3D spheroids. The real-time label-free monitoring of cell invasion afforded by the impedance sensor system allowed for precise and quantitative assessment, offering insight into the temporal dynamics of cell responses. Our results underscore the potential of impedance sensing as a valuable tool for studying the intricate interplay between cells and their microenvironment in three-dimensional settings. This integrated methodology holds promise for advancing anti-metastasis drug screening strategies. Moreover, the successful validation of the DFO drug as an inhibitor of 3D spheroid invasion highlights the translational potential of our findings. By bridging the gap between traditional 2D cell culture models and more complex 3D systems, our study establishes a robust foundation for future investigations into anti-metastasis drug screening.

## Data Availability

The raw data supporting the conclusions of this article will be made available by the authors, without undue reservation.
